# Identifying MicroRNA Markers That Predict COVID-19 Severity Using Machine Learning Methods

**DOI:** 10.3390/life12121964

**Published:** 2022-11-23

**Authors:** Jingxin Ren, Wei Guo, Kaiyan Feng, Tao Huang, Yudong Cai

**Affiliations:** 1 School of Life Sciences, Shanghai University, Shanghai 200444, China; 2 Key Laboratory of Stem Cell Biology, Shanghai Jiao Tong University School of Medicine (SJTUSM) & Shanghai Institutes for Biological Sciences (SIBS), Chinese Academy of Sciences (CAS), Shanghai 200030, China; 3 Department of Computer Science, Guangdong AIB Polytechnic College, Guangzhou 510507, China; 4 Bio-Med Big Data Center, CAS Key Laboratory of Computational Biology, Shanghai Institute of Nutrition and Health, University of Chinese Academy of Sciences, Chinese Academy of Sciences, Shanghai 200031, China; 5 CAS Key Laboratory of Tissue Microenvironment and Tumor, Shanghai Institute of Nutrition and Health, University of Chinese Academy of Sciences, Chinese Academy of Sciences, Shanghai 200031, China

**Keywords:** COVID-19, SARS-CoV-2, MicroRNA, feature analysis, biomarker, rules

## Abstract

Individuals with the SARS-CoV-2 infection may experience a wide range of symptoms, from being asymptomatic to having a mild fever and cough to a severe respiratory impairment that results in death. MicroRNA (miRNA), which plays a role in the antiviral effects of SARS-CoV-2 infection, has the potential to be used as a novel marker to distinguish between patients who have various COVID-19 clinical severities. In the current study, the existing blood expression profiles reported in two previous studies were combined for deep analyses. The final profiles contained 1444 miRNAs in 375 patients from six categories, which were as follows: 30 patients with mild COVID-19 symptoms, 81 patients with moderate COVID-19 symptoms, 30 non-COVID-19 patients with mild symptoms, 137 patients with severe COVID-19 symptoms, 31 non-COVID-19 patients with severe symptoms, and 66 healthy controls. An efficient computational framework containing four feature selection methods (LASSO, LightGBM, MCFS, and mRMR) and four classification algorithms (DT, KNN, RF, and SVM) was designed to screen clinical miRNA markers, and a high-precision RF model with a 0.780 weighted F1 was constructed. Some miRNAs, including miR-24-3p, whose differential expression was discovered in patients with acute lung injury complications brought on by severe COVID-19, and miR-148a-3p, differentially expressed against SARS-CoV-2 structural proteins, were identified, thereby suggesting the effectiveness and accuracy of our framework. Meanwhile, we extracted classification rules based on the DT model for the quantitative representation of the role of miRNA expression in differentiating COVID-19 patients with different severities. The search for novel biomarkers that could predict the severity of the disease could aid in the clinical diagnosis of COVID-19 and in exploring the specific mechanisms of the complications caused by SARS-CoV-2 infection. Moreover, new therapeutic targets for the disease may be found.

## 1. Introduction

Since 2019, the novel coronavirus disease caused by severe acute respiratory syndrome coronavirus 2 (SARS-CoV-2) has had a severe negative impact on the world’s economy and public health. As of 23 June 2022, more than 539 million patients have been diagnosed with the infection, and more than 6.3 million deaths have been reported worldwide [1]. The SARS-CoV-2 infection causes symptoms that vary widely among individuals and could cause symptoms ranging from asymptomatic to fever and cough with mild symptoms to severe respiratory impairment that could even lead to death [2,3,4,5]. The course of the disease cannot be accurately predicted. Given the uncertain clinical response to COVID-19 and the wide range of complications, novel biomarkers that could predict the severity of the disease and help in the clinical diagnosis of COVID-19 could help us to explore the specific mechanisms of the complications caused by the SARS-CoV-2 infection and find new therapeutic targets.

To date, studies have compared the hematological and biochemical parameters and inflammatory biomarkers in COVID-19 patients of different severity levels and have found changes in various parameters, including lymphocytes, the C-reactive protein, albumin, and small molecules, such as coagulation factors and various inflammatory chemokines, which are more easily observed in severely and critically ill patients [5,6,7,8,9,10,11]. These parameters may be related to more complex complications in the later stages of the disease. To better explore the role played by various parameters in the immune system during COVID-19, we screened miRNAs as new biomarkers to identify patients with different COVID-19 severities.

MicroRNAs (miRNAs) are a class of highly conserved short noncoding RNA molecules with regulatory roles not directly involved in protein coding. However, they regulate gene expression at the posttranscriptional level by specific complementary binding to mRNAs. Many miRNAs were identified as biomarkers for many diseases, including cancer and cardiovascular diseases [12,13]. Among them, the role of miRNAs in the process of viral infection was highlighted in the diseases caused by exogenous viruses. Some miRNAs could inhibit viral infection through channels, such as the regulation of viral protein receptors and chemokine expression [14,15], whereas partially dysregulated miRNAs cause inflammation and disrupt autophagy [16,17], thereby leading to cellular damage and promoting viral replication. Changes in miRNA expression profiles in COVID-19 patients were identified in previous studies [18,19]. Some of these differentially expressed miRNAs are involved in the antiviral effects of the SARS-CoV-2 infection [20,21,22,23,24]. Some help with viral entry into host cells, as well as viral protein synthesis and assembly [25,26]. Such differences in miRNA expression could also be observed in COVID-19 patients of different severity levels [27]. Thus, screening miRNAs as biomarkers to help identify COVID-19 patients or to characterize COVID-19 severity could facilitate more accurate clinical diagnoses and explore the potential regulatory mechanisms in the course of the SARS-CoV-2 infection.

In the current study, we collected data on the plasma miRNA levels in COVID-19 patients from two previous studies [28,29]. Zeng et al. performed high throughput sequencing to detect the miRNAs in the plasma samples collected from patients with different symptoms of COVID-19 and identified 2336 known miRNAs and 361 novel miRNAs, which were associated with the various clinical presentations and viral persistence levels of COVID-19. Gustafson et al. evaluated miRNA expression and co-regulatory network generation and found that the transcriptome of critically ill COVID-19 patients was significantly deregulated, largely unlike that of SARS-CoV-2-negative patients in the ICU. Integrating the results reported in the above two studies would yield a more accurate miRNA profile of COVID-19 patients, because both studies examined the miRNA expression of patients with various COVID-19 symptoms at the plasma level. Along with machine learning methods, we were able to identify the biomarkers that distinguish the severity of COVID-19 with a more comprehensive miRNA profile. In detail, four feature selection methods, including the least absolute shrinkage and selection operator (LASSO) [30,31], the light gradient boosting machine (LightGBM) [32], the Monte Carlo feature selection (MCFS) [33], and the minimum redundancy maximum relevance (mRMR) [34], were used to evaluate the features associated with the infection status, generating four feature lists that ranked the features according to their importance to the prediction. Then, optimal prediction models based on four classification algorithms (decision tree (DT) [35], k-nearest neighbor (KNN) [36], random forest (RF) [37], and support vector machine (SVM) [38]) were built by applying the incremental feature selection (IFS) method [39] to each feature list. The features used in these models were deemed essential for distinguishing the COVID-19 symptoms and could be novel biomarkers. Furthermore, some interesting decision rules were obtained with the DT, which could indicate special miRNA patterns for different COVID-19 symptoms. The usage of multiple methods could overcome the data preference for specific algorithms, extract more reliable features, and establish more accurate decision rules for quantitative differentiation. The set of rules could help to distinguish between COVID-19 patients, SARS-CoV-2-negative patients with upper respiratory symptoms, and the healthy population, thereby further determining the disease severity of individuals, especially the COVID-19 patients. These identified biomarkers and rules may help in the clinical diagnosis of COVID-19 and uncover its disease mechanisms.

## 2. Materials and Methods

### 2.1. Data and Preprocessing

The data on the plasma miRNA levels of COVID-19 patients from two previous studies (GSE166160 and GSE178246) were combined to create a synthesized dataset [28,29]. The study by Zeng et al. (GSE166160) involved 66 healthy controls, 16 asymptomatic COVID-19 patients, and 149 symptomatic COVID-19 patients, including 66 with moderate COVID-19 and 83 with severe COVID-19. The study by Gustafson et al. (GSE178246) involved 30 SARS-CoV-2-negative patients with mild upper respiratory tract symptoms, 14 patients with mild COVID-19, 15 patients with moderate COVID-19, 54 patients with severe COVID-19, and 31 SARS-CoV-2-negative patients from the ICU with upper respiratory tract symptoms. Generally, asymptomatic COVID-19 patients could be categorized as mild COVID-19 patients. In addition, we renamed two classes of samples; the SARS-CoV-2-negative patients with mild upper respiratory tract symptoms and SARS-CoV-2-negative patients from the ICU with upper respiratory tract symptoms were renamed as non-COVID-19 patients with mild symptoms, and non-COVID-19 patients with severe symptoms, respectively. Finally, 30 patients with mild COVID-19 symptoms, 81 patients with moderate COVID-19 symptoms, 30 non-COVID-19 patients with mild symptoms, 137 patients with severe COVID-19, 31 non-COVID-19 patients with severe symptoms, and 66 healthy controls were identified in this study. In terms of the features, a total of 1444 miRNAs shared in two datasets, GSE166160 and GSE178246, were used as the features of this study.

### 2.2. Feature Ranking Algorithms

Four powerful algorithms: LASSO [30,31], LightGBM [32], MCFS [33], and mRMR [34], were employed to assess the miRNA features and rank them in lists. Their brief descriptions are as follows.

#### 2.2.1. LASSO

In LASSO, the variables were selected and compressed to reduce the feature dimension based on the linear regression models [30,31]. The L1 paradigm was used to create a penalty function that selectively removed the low-correlation variables by penalizing features with higher coefficients and greater prediction errors. Through this process, fewer feature variables were included in the model, and the feature dimension was effectively reduced. The importance of a feature was the absolute value of its coefficient. The features could then be ranked based on their coefficients. The LASSO program from Scikit-learn was run with the default paraments.

#### 2.2.2. LightGBM

LightGBM is a well-known boosting learning machine that combines many weak classifiers to achieve a single strong one [32]. It is an improved version of the gradient boosting decision tree (GBDT), which recurrently fits a new DT, whose residual is approximated by the negative gradient of the loss function of the current DT. It has the following advantages: it is fast, uses less memory, has higher accuracy, can support parallel learning, and can handle large quantities of data. In addition to classification, LightGBM sorts features based on their importance, as quantified by the number of times they are selected for building DTs. More frequently used features are ranked higher. The LightGBM program was implemented through a Python module called lightGBM. The default parameters were adopted to execute this program.

#### 2.2.3. MCFS

MCFS involves the construction of several bootstrap sets and randomly selects some feature sets; it is a powerful and widely used method for selecting features [33,40,41]. Some features are randomly selected as a feature subset, which is used to re-represent the new training samples. M bootstrap sets are constructed from the new training samples to build M decision trees. This process is repeated T times, resulting in M × T trees. According to its involvement in building M × T trees, a feature is rated according to its relative importance (*RI*):

(1) $$RI_{g}=\sum_{\tau =1}^{MT}{\left(wAcc\right)}^{u}\sum_{n_{g}\left(\tau \right)}IG\left(n_{g}\left(\tau \right)\right){\left(\frac{no.in\;n_{g}\left(\tau \right)}{no.in\;\tau }\right)}^{v},\;$$

where wAcc is the weighted accuracy. $IG\left(n_{g}\left(\tau \right)\right)$ stands for the information gain (*IG*) of $n_{g}\left(\tau \right)$ (a DT node n with the attribute g). $no.in\;n_{g}\left(\tau \right)\;$ stands for the number of samples in $n_{g}\left(\tau \right)$. no.in τ stands for the sample sizes in the tree root. u and v are two settled positive integers.

This study used the MCFS program downloaded from http://www.ipipan.eu/staff/m.draminski/mcfs.html (accessed on 4 June 2019). It was performed using the default parameters.

#### 2.2.4. mRMR

Using mRMR, the features were ranked based on the maximum relevance with the class variable and minimum redundancy between the features [34]. Mutual information (MI) could be defined as follows:

(2) $$I\left(x,y\right)=\iint p\left(x,y\right)\log\frac{p\left(x,y\right)}{p\left(x\right)p\left(y\right)}dxdy,\;$$

where $p\left(x,y\right)$ represents the joint probabilistic density of x and y, and $p\left(x\right)$, and $p\left(y\right)$ represent the marginal probabilistic densities of x and y, respectively.

Let Ω represent the feature set that includes the features to be selected.  Ω′ denotes the feature set that includes the features that are already selected. $f_{i}$ or $f_{j}$ represent a feature in Ω or  Ω′, and C is the class label. The mRMR function is defined as follows:

(3) $$argmax\left(f_{i}\right)=I\left(f_{i},\;C\right)-\frac{1}{\left|\;\Omega \prime \right|}\sum_{f_{j}\in \;\Omega \prime }I(f_{i,\;\;}f_{j}),\;$$

where $I\left(f_{i},\;C\right)$ indicates the MI between $f_{i}$ and the class variable *C* and $I\left(f_{i},\;f_{j}\right)\;$ represents MI between $f_{i}$ and $f_{j}$.

The mRMR program was obtained from http://home.penglab.com/proj/mRMR/ (accessed on 2 May 2018) for this work and run with the default settings.

### 2.3. Incremental Feature Selection

The IFS method was utilized to find the appropriate number of features to build the optimal models [39,42,43]. The procedure of the IFS method could be divided as follows:

1. Each feature matrix was constructed using the top $n\;\left(n=1,\;2,\;\ldots ,\;k\right)$ features from the four feature ranking algorithms, where k is the total number of features;
2. The 10-fold cross-validation was performed on each feature matrix to evaluate the performance of the classification model.
3. The most effective classification model and its feature subset were selected for each of the four feature rankings.

IFS was used to identify the optimal subset of features for the four feature ranking algorithms, and Venn diagrams were drawn to examine their intersections.

### 2.4. Synthetic Minority Oversampling Technique

Considering that the sample sizes of each class were different, the synthetic minority oversampling technique (SMOTE) method was used to balance the dataset [44,45]. This approach creates an equal number of samples for each class by linearly synthesizing new sample data using a randomly selected sample and one of its k-nearest neighbors. These additional data were added to train the classification model and improve its performance during the 10-fold cross-validation test. We used the SMOTE tool from https://github.com/scikit-learn-contrib/imbalanced-learn (accessed on 24 March 2020) with default parameters.

### 2.5. Classification Algorithm

To make the predictions, four classification models were constructed, as follows: DT [35], KNN [36], SVM [38], and RF [37], which are widely used in bioinformatics [46,47,48,49,50,51,52]. The DT is a flowchart-like tree structure with logical operations at its internal nodes. A leaf node holds a class label. The predictions are created by starting at the root node and sorting the data down the tree to a leaf node according to the outcome of the test defined for each branch [35]. In the KNN classification, new samples are assigned to their classes based on the voting of their k-nearest neighbor samples [36]. The SVM is a machine learning model based on statistical learning theory that linearly separates the data by locating the maximum margin separating hyperplane; it could map data into a high-dimensional space using a kernel function [38]. RF is a machine learning algorithm that constructs a number of tree classifiers based on the Bagging algorithm; the data are classified by majority voting [37]. These algorithms were implemented by packages collected in Scikit-learn, which were directly employed in this study. For convenience, they were executed with their default parameters.

### 2.6. Performance Evaluation

The weighted *F*1 was mainly used to evaluate the performance of the classification models. To compute the weighted *F*1, the *F*1 score for each class should be computed first. For the *i*-th class, its *F*1 score is defined as:

(4) $$F1\;score_{i}=\frac{2\times Precision_{i}\times Recall_{i}}{Precision_{i}+Recall_{i}},\;$$

(5) $$Precision_{i}=\frac{TP_{i}}{TP_{i}+FP_{i}},\;$$

(6) $$Recall_{i}=\frac{TP_{i}}{TP_{i}+FN_{i}},\;$$

where *TP_i_*, *FP_i_* and *FN_i_* denote true positive, false positive, and false negative for the *i*-th class. The weighted *F*1 is defined as the weighted average of the *F*1 scores on all classes, where the weight is the proportion of the number of samples in one class to the total number of samples. The direct average of the *F*1 scores defines another widely used measurement, macro *F*1. In addition, we also used two other measurements: prediction accuracy (ACC) and the Matthews correlation coefficient (*MCC*). The ACC is defined as the proportion of correctly classified samples. For the *MCC*, two binary matrices *X* and *Y* should be constructed first, which denote the actual and predicted classes of all samples. Then, it could be computed using the following formula:

(7) $$MCC=\frac{cov\left(X,Y\right)}{\sqrt{cov\left(X,X\right)cov\left(Y,Y\right)}},\;$$

where *cov*(.) represents the covariance of the two matrices.

## 3. Results

**Figure 1:**
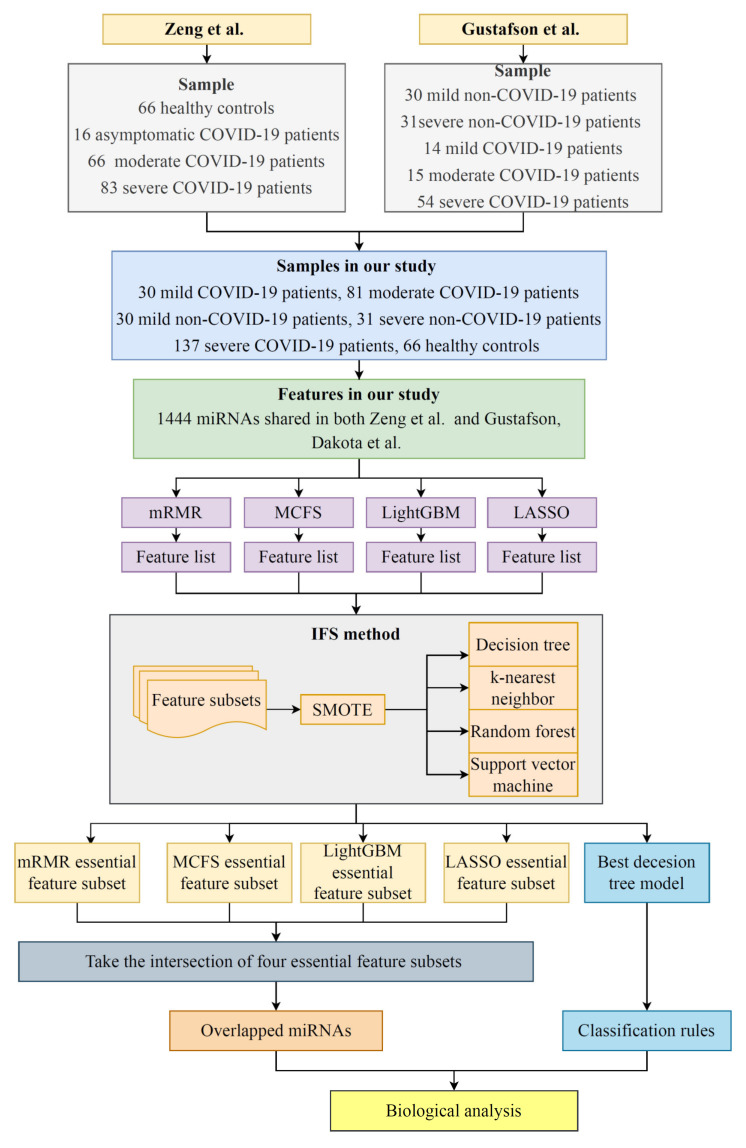
Flow chart of the entire analytical process. The 1444 miRNAs integrated from Zeng et al.’s and Gustafson et al.’s studies [28,29] were ranked according to the feature importance using four feature ranking algorithms, namely, LASSO, LightGBM, mRMR, and MCFS. Then, four ordered feature lists were fed into the IFS framework, and efficient classification models were constructed. The essential feature subset in each feature list and the classification rules were extracted. We intersected the subsets to obtain the miRNAs that recurred in multiple subsets. Finally, a biological analysis of the overlapping miRNAs and classification rules was performed.

The key miRNAs and classification rules were extracted to help us distinguish between the COVID-19 patients, the SARS-CoV-2-negative patients with upper respiratory symptoms, and the healthy population, as well as to further determine the severity of the disease in individuals, especially the COVID-19 patients. The overall computational framework is shown in Figure 1. Next, we provided details of the results at each step.

### 3.1. Results of the Feature Ranking Algorithms

The results of ranking 1444 miRNA features using LASSO, LightGBM, MCFS, and mRMR are shown in Table S1. In the classification process, the features that participated in the top ranked list are important. The biological significance of these features and their significance as classification features are discussed in Section 4.

### 3.2. IFS Results and Feature Intersections

**Figure 2:**
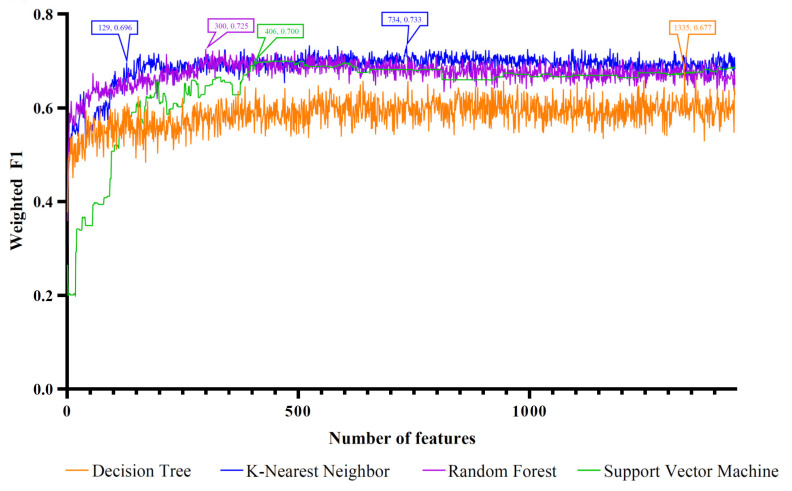
IFS curves based on the feature list generated with LASSO and the four classification algorithms. Four classification algorithms yielded the highest weighted F1 values when the top 1335, 734, 300, and 406 features were used. The KNN also provided a high performance based on the top 129 features.

**Figure 3:**
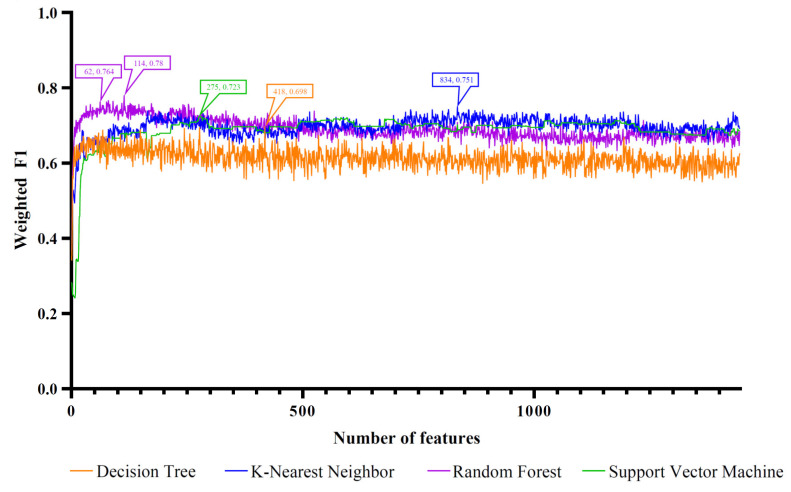
IFS curves based on the feature list generated with LightGBM and the four classification algorithms. Four classification algorithms yielded the highest weighted F1 values when the top 418, 834, 114, and 275 features were used. The RF also provided a high performance based on the top 62 features.

**Figure 4:**
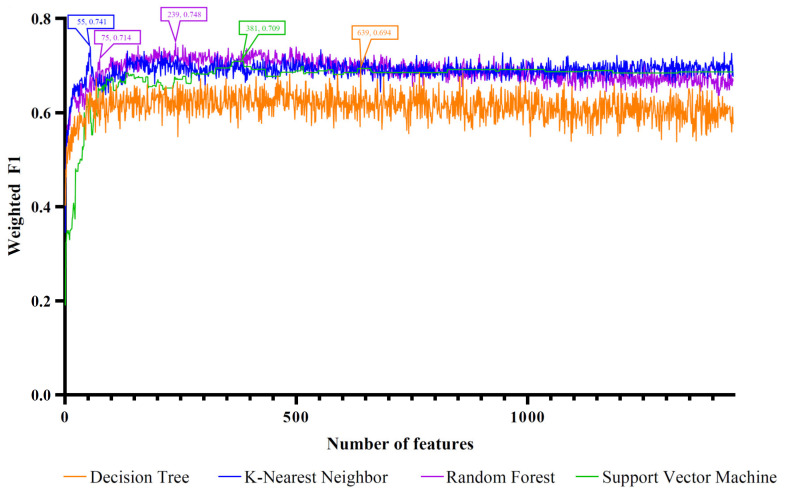
IFS curves based on the feature list generated with MCFS and the four classification algorithms. Four classification algorithms yielded the highest weighted F1 values when the top 639, 55, 239, and 381 features were used. The RF also provided a high performance based on the top 75 features.

**Figure 5:**
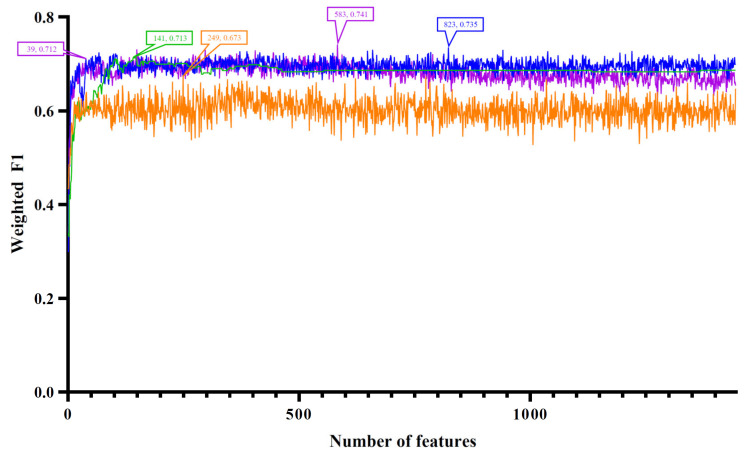
IFS curves based on the feature list generated with mRMR and the four classification algorithms. Four classification algorithms yielded the highest weighted F1 values when the top 249, 823, 583, and 141 features were used. The RF also provided a high performance based on the top 39 features.

The four ordered miRNA lists created with the feature ranking algorithms were fed into the IFS method one by one, which incorporated four classification algorithms. With the IFS method, we acquired feature subsets of varied sizes by increasing the feature numbers in order. The models built on different feature subsets were compared for the assessment of their classification performance by using the weighted F1. The IFS curve was produced by charting the weighted F1 on the y axis and the number of features on the x axis. Different curves are drawn for various classification algorithms. The four feature ranking methods corresponded to four sets of IFS curves, as shown in Figure 2, Figure 3, Figure 4 and Figure 5. The detailed results are presented in Table S2.

**Figure 6:**
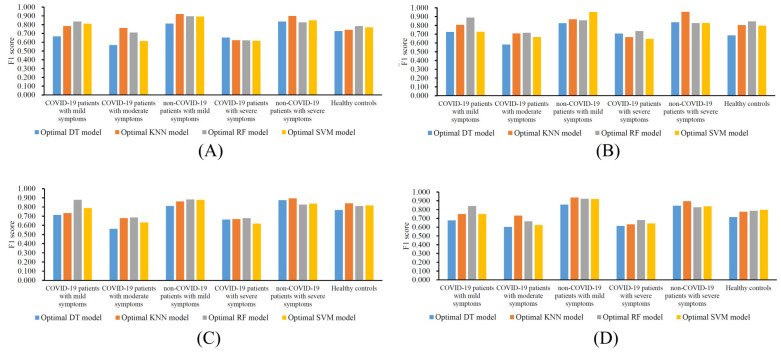
Performance of the optimal models built on different feature lists in six classes. (**A**) Feature list generated with LASSO, (**B**) feature list generated with LightGBM, (**C**) feature list generated with MCFS, (**D**) feature list generated with mRMR.

For the feature list generated by LASSO, the IFS curves corresponding to the four classification algorithms are illustrated in Figure 2. It can be seen that the highest weighted F1 values for DT, KNN, RF, and SVM were 0.677, 0.733, 0.725, and 0.700, respectively. Such performance was obtained using the top 1335, 734, 300, and 406 features in the list. Accordingly, the optimal DT, KNN, RF, and SVM models were constructed with these features. Their detailed overall performance is listed in Table 1, and their performance in each class is shown in Figure 6A. Generally, the optimal KNN model was the best among all of the optimal models. The features used in this model (the top 734 features in the list) constituted the optimal feature set of LASSO.

For the feature list produced with LightGBM, four IFS curves are illustrated in Figure 3. DT, KNN, RF, and SVM generated the highest weighted F1 values of 0.698, 0.751, 0.780, and 0.723, respectively, when the top 418, 834, 114, and 275 features in the list were adopted. Likewise, four optimal models could be set up with these features. The detailed performance of these models can be found in Table 1 and Figure 6B. Evidently, the optimal RF model gave the best performance. Thus, the features used in this model (the top 114 features in the list) comprised the optimal feature set of LightGBM.

For the third feature list yielded with MCFS, the IFS curves are shown in Figure 4. The highest weighted F1 values for the four classification algorithms were 0.694, 0.741, 0.748, and 0.709, respectively. The top 639, 55, 239, and 381 features in the list were used to achieve such performance. With these features, the optimal DT, KNN, RF, and SVM models were built. Table 1 and Figure 6C list their detailed performance, from which we can see that the optimal RF model was the best. Accordingly, the optimal feature set of MCFS included the features used in this model (the top 239 features in the list).

For the last feature list generated with mRMR, Figure 5 shows the IFS curves. The highest weighted F1 values for DT, KNN, RF, and SVM were 0.673, 0.735, 0.741, and 0.713, respectively, which were obtained using the top 249, 823, 583, and 141 features in the list. Then, the optimal DT, KNN, RF, and SVM models were set up based on these features. Their detailed performance is listed in Table 1 and Figure 6D. The optimal RF model was still the best, and its used features (the top 583 features in the list) constituted the optimal feature set of mRMR.

**Figure 7:**
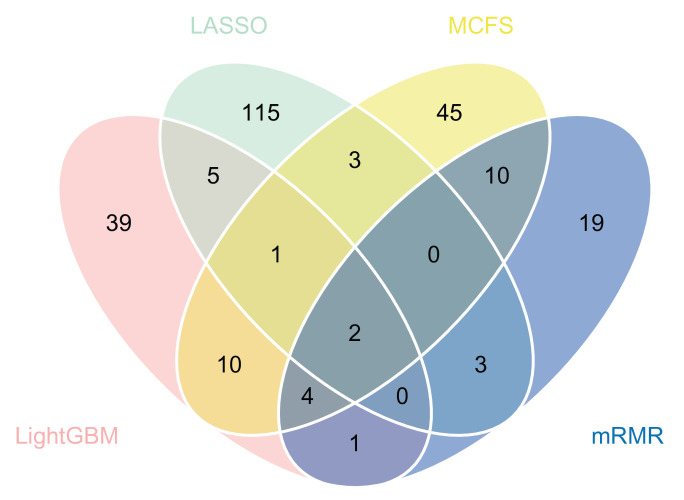
Venn diagram showing the intersections of the essential feature sets of LASSO, LightGBM, MCFS, and mRMR. The overlapping circles indicate the miRNAs that appeared in multiple sets.

Four optimal feature sets were constructed from four feature lists. However, there were many features in each set, which was not easy for extensive analyses. Thus, we tried to find the most important features of each feature set. By checking the IFS results on each feature list (Table S2), the model with the same classification algorithm could provide relatively high performance when fewer features were employed. For example, when the top 129 features in the optimal feature set of LASSO were considered, the KNN could yield a weighted F1 of 0.696, which was only a little lower than that of the optimal KNN model. These 129 features were much more important than the other 605 (734–129) features in the optimal feature set, as the rest of the features could increase the weighted F1 by only 0.037. The overall performance of the KNN model with the top 129 features is listed in Table 2. Clearly, its performance was slightly lower than that of the optimal KNN model (Table 1). We termed the set consisting of these features as the essential feature set of LASSO. Using similar arguments, the essential feature set of LightGBM, MCFS, and mRMR could be determined, which contained the top 62, 75, and 39 features in the corresponding optimal feature set. Table 2 shows the performances of the models with these essential features. They were all slightly inferior to the optimal models. As the feature lists were generated with different feature ranking algorithms, which have different principles, the analysis of all the essential feature sets was helpful in extracting the miRNA biomarkers as completely as possible. Thus, we intersected the essential feature sets of LASSO, LightGBM, MCFS, and mRMR to obtain the overlapping miRNAs that appeared in multiple sets. By combining the above feature sets, 275 features were obtained. Their distribution on four essential feature sets is illustrated with a Venn diagram, as shown in Figure 7. The detailed results can be found in Table S3. Several miRNAs appeared in multiple sets, suggesting that they may play important roles in differentiating patients with different severities of COVID-19. The biological significance of these miRNAs is discussed in Section 4.

### 3.3. Classification Rules

The classification rules were extracted using the white-box model DT that visualized the classification process. The IFS results showed that the DT achieved optimal classification performance when the numbers of features were 1335, 418, 639, and 249. The classification rules were extracted based on the optimal DT model under the feature numbers mentioned above. From the optimal DT model built on the list generated with LASSO, 63 classification rules were obtained. For the other three DT models, they provided 69, 63, and 70 classification rules, respectively. Each rule is comprised of miRNA features and their expression values. It describes how the feature’s high or low expression value affects the ability to distinguish among the various classes of samples. The specific classification rules are shown in Table S4. The details of the quantitative rules are presented in Section 4.

## 4. Discussion

We integrated data from two studies on the miRNA levels in the plasma of COVID-19 patients [28,29] and obtained multiple feature lists based on four feature ranking algorithms. The corresponding classification models were built. The features extracted from these feature lists may be an excellent set of potential biomarkers that could reveal differences between the different symptoms of infection and between the different levels of severity after being infected. These biomarkers could help us to analyze the disease in patients with COVID-19 and reveal the mechanisms of miRNA regulation under SARS-CoV-2 infection. We selected some top features (miRNAs) that appeared in multiple lists for discussion. The representative miRNAs and their predictive roles were summarized and presented in Table 3. The analyses on them were as follows.

### 4.1. Analysis of the Key Biomarkers

The first feature analyzed was miR-24-3p. This feature appeared in the list of features of four methods and was considered a valid biomarker. miR-24-3p acts directly on the 3’ UTR of neuropilin-1 (NRP) mRNA [85], which expresses a non-tyrosine kinase surface glycoprotein in vertebrates. The NRP-1 transmembrane isoform serves as a target for direct binding to the S1 polypeptide after SARS-CoV-2 protein hydrolysis [86], thereby increasing the enhanced infectivity during acute severe SARS-CoV-2 infection [87]. NRP-1 uses vascular endothelial growth factor (VEGF)165 as a bridge to the VEGF receptor 2 to induce angiogenesis [88]. VEGF165 binds to the b1 structural domain of NRP-1, which is also the site of SARS-CoV-2 binding to NPR-1. SARS-CoV-2 infection leads to the dysregulation of vascular and coagulation functions. In addition, the NRP-1 transmembrane isoform contributes to the infectivity of SARS-CoV-2 by acting as a target for direct binding to the S1 polypeptide after the hydrolysis of SARS-CoV-2 proteins [86,87]. miR-24-3p could directly inhibit the replication of the SARS-CoV-2 virus [53]. The differential expression of miR-24-3p was found in patients with sequelae of acute lung injury caused by severe COVID-19 [54]. These results suggested the research potential of miR-23-3p.

The second feature analyzed was miR-93-3p. This feature was also mentioned in the list of four features and is a highly validated biomarker. No studies have elaborated on a direct relationship between this feature and COVID-19. miR-93-3p targets toll-like receptor 4 (TLR4) and inhibits its expression through a posttranscriptional regulatory mechanism [89], which accelerates the production of proinflammatory factors through the NF-κB pathway [90,91,92]. The upregulated expression of miR-93-3p was observed in macrophages during HIV infection, suggesting the potential role of miR-93-3p in viral-related immune responses [93]. miR-93-3p has serious effects on the cardiovascular system, triggering symptoms that lead to a variety of conditions, including myocarditis [55] and the development of cytokine storms [56]. miR-93-3p might play an important role in COVID19-triggered tissue damage.

The features discussed next were all present in the list of features of the three methods. miR-148a-3p was the third feature analyzed. One study reported that miR-148a-3p regulates the Ras/MAPK/Erk signaling pathway to suppress cancer by targeting Son of sevenless 2 (one of the guanylate nucleotide exchange factors) [94] and ultimately affects B cell differentiation by targeting transcription factor mRNAs, such as Bach2, Mitf, and others [95,96]. In the studies on the differentiation of ICU patients from ward patients, miR-148a-3p was selected for the assessment of COVID-19 severity [57]; the mechanisms involved have not been elucidated yet. In addition, miR-148a was differentially expressed against SARS-CoV-2 structural proteins [57,58], which may allow these structural proteins to escape miRNA-mediated repression and aid in the spread of the virus in the early stages of infection.

The fourth feature analyzed was miR-139-5p. miR-139-5p was upregulated in SARS-CoV-2 infected cells [59] and was associated with COVID-19 severity [60]. MiR-139-5p could reduce the production of proinflammatory cytokines and chemokines by targeting genes, such as MyD88, c-FOS, and Rap1b, and mediating NF-κB and STAT3 signaling pathways [97,98,99,100]. This result might be explained by the overexpression of miR-139-5p in COVID-19 patients, which is the organism’s stress response to infectious inflammation.

The fifth feature analyzed was miR-199a-5p. Few reports have been published on COVID-19 with miR-199a-5p. In a study comparing the bronchial aspirate in critically ill COVID-19 patients versus non-COVID-19 patients, multiple miRNAs, including miR-199a-5p, were identified; these could be used as markers to distinguish between the two samples [61]. The miR-199a-5p could reportedly inhibit the secondary envelope of the virus and exert antiviral effects by inhibiting the target ARHGAP21, which is a Golgi-localized GTPase-activating protein of Cdc42 [62]. miR-199a-5p possibly plays a role in COVID-19 infection.

The sixth signature analyzed was miR-17-3p, which is a signature of several potential targets, such as GFRA2 [101], PAR4 [102], and TIMP3 [103]. These targets are associated with cell proliferation and development. miR-17-3p could target the NIBP gene to inhibit NF-κB activation, thereby suppressing inflammation [104]. In COVID-19 patients, the miR-17-3p expression was proportional to the grading of infection [63]. miR-17-5p, another product of the miR-17 cluster, was also screened as an anti-SARS-CoV2 miRNA; its expression was significantly downregulated in patients [54,64,65]. SARS-CoV-1 expands replication by hijacking miR-17 [66]. Taken together, the production levels of miR-17 clusters are highly correlated with COVID-19 infection.

The last feature analyzed was miR-200c-3p. The direct target of miR-200c-3p is angiotensin-converting enzyme 2 (ACE2), which is a functional receptor necessary for SARS-CoV-2 entry into cells [67,68]. miR-200c-3p has an inverse regulatory effect on ACE2 expression. The differential expression of miR-200c-3p was observed in patients with different severity levels [69,70,71]. In addition, miR-200c-3p directly targets IL-8 [105], which is a proinflammatory chemokine and influences the development of cytokine storms [106].

**Figure 8:**
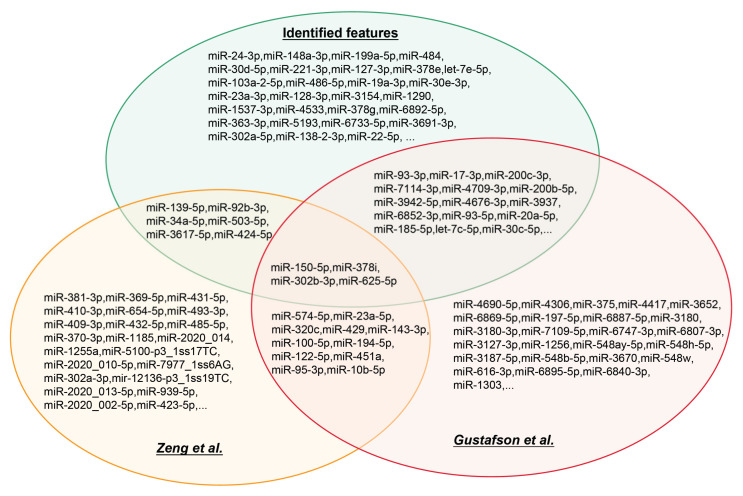
Venn diagram showing the comparison of the miRNAs associated with COVID-19 identified in this study and Zeng et al. and Gustafson et al.’s studies [28,29].

Zeng et al. [29] identified 85 differentially expressed miRNAs (DE-miRNA) associated with COVID-19 and finally screened six miRNAs for use in differentiating SARS-CoV-2 infected individuals from healthy controls. The cross-sectional study by Dakota Gustafson et al. [28] examined the differentially expressed miRNAs between COVID-19 survivors and non-survivors. SARS-CoV-2 demonstrated differentially expressed miRNAs between negative individuals. The current study integrated them to obtain a richer miRNA profile of patients with different levels of COVID-19 by using four machine learning algorithms to obtain a ranked list of miRNA correlations. A total of 257 biomarkers were obtained for differentiating the samples, some of which could be supported by the recent publications described above. A systematic comparison between the COVID-19-related miRNAs identified in this study and the differentially expressed miRNAs reported by Zeng et al.’s and Gustafson et al.’s studies [28,29] is shown in Figure 8. Several miRNAs identified in this study were also reported in Zeng et al.’s and Gustafson et al.’s studies, indicating the reliability of our results. Furthermore, some exclusive miRNAs were discovered by our study, which could be novel biomarkers.

### 4.2. Analysis of the Classification Rules

As previously described, the top features obtained could participate in the classification of the samples based on the support of existing publications. Accordingly, we further established four quantitative rule sets to determine the infection status of the distinguished samples by using the newly proposed computational method. Each set of rules could help to distinguish between COVID-19 patients, SARS-CoV-2-negative patients with upper respiratory tract symptoms, and the healthy population. It could further determine the severity of infection in infected patients, especially COVID-19 patients. In conjunction with the purpose of this study, the following discussion focuses on the parameters used to identify patients with severe COVID-19. These parameters are derived from the four methods of the high accuracy top rules. We discuss the parameters in order according to their number of occurrences.

First, a parameter, miR-6750-5p, appeared in the rules of all four methods. miR-6750-5p was downregulated in all four sets of rules, which indicates severe COVID-19. miR-6750-5p, as a late discovered miRNA, has been poorly documented, and no report describes its direct relationship with COVID-19. A study on the miRNA profile of immunoglobulin A nephropathy of different severity levels claimed that the expression level of miR-6750-5p was significantly downregulated in the more severe forms of nephritis [72]. We inferred that the downregulation of this parameter in the rule might be associated with the high inflammation caused by severe COVID-19. In conclusion, miR-6750-5p is a very promising biomarker.

The second parameter discussed (miR-93-5p) is mentioned in the rules of LASSO, MCFS, and mRMR. miR-93-5p requires a lower level to indicate severe COVID-19. According to publications, miR-93-5p expression could inhibit the expression of chemokines, such as IL-6 and IL-8 [73,107,108,109]. These chemokines play a key role in the “cytokine storm” and inflammation that occurs in patients with severe COVID-19 and reflect the severity of the infection [110,111,112]. Considering the report that miR-93-5p has a protective effect on inflammation [73], we suggested that miR-93-5p could potentially serve as a biomarker for recognizing patients and could be used as a therapeutic target.

The parameters that appeared in both LASSO and MCFS methods are analyzed next. The first parameter discussed is miR-34a-5p. The downregulation of this parameter indicates severe COVID-19. The enrichment results on miR-34a-5p and its associated miRNAs showed that they are involved in endothelial cell function, inflammation, and the pathways of viral diseases [74]. miR-34a-5p could inhibit the NF-κB pathway to attenuate the inflammatory response [75,76] and alleviate lung injury [77]. miR-34a-5p expression was significantly reduced in the lung tissues or airway samples of COVID-19 patients [61,74], contrary to the results obtained with plasma samples. This finding might be related to the progression of SARS-CoV-2 infection or the immune response in different tissues of the organism. miR-29b-2-5p was also mentioned by both approaches, showing upregulation in the rules for identifying severe COVID-19. Only one study showed its possible involvement in cytokine overproduction in COVID-19 patients [78]. miR-6762-3p, miR-4709-3p, miR-6791-5p, and miR-4685-3p were also characterized by the common screening of LASSO and MCFS approaches but as late discovered miRNAs, few relevant literature reports are available, and the roles and mechanisms for these miRNAs still need further experimental exploration.

In addition to the above parameters that appeared in multiple methods, each method also screened for its own unique features. miR-429 is a parameter that belongs only to the LASSO method. miR-429 is a member of the miR-200 family. Several members of this family are involved in cytokine regulation. miR-429 could reduce the production of inflammatory cytokines and alleviate lung injury [79]. Another study also raised the possibility of drug-induced miR-429 downregulation for myocarditis [80]. miR-429 is downregulated in the rule, possibly in the response of the organism to inflammation in patients with severe COVID-19. miR-205-5p is a parameter that belongs only to the LightGBM approach. Increased levels of proinflammatory factors, such as NF-κB signaling indicator p65 and IL-6, were observed when miR-205-5p was inhibited [81]. In viral infection studies, miR-205-5p was used as a biomarker for the detection of influenza B [82]; miR-205-5p was differentially expressed in the case of mixed hepatitis C and HIV infections [83]. In the rule, miR-205-5p expression was downregulated. miR-873-5p is a parameter that belongs only to the MCFS approach and shows downregulation in the rule. miR-873-5p is regulated and mediated by IL-17; treatment with the anti-miR-873-5p protected against inflammation-induced injury [84]. The mRMR approach top rules have fewer parameters specific to the mRMR approach and are mostly newly discovered miRNAs. Few studies have been reported. Its role in the infection of SARS-CoV-2 needs further investigation.

In conclusion, all four selected methods yielded quantitative rules with significant effects and strong classification abilities, and each method has its own characteristic parameters in its rules. The top quantitative rules could all be supported by recent publications, thereby verifying the reliability of the obtained rules.

### 4.3. The Advantages and Limitations of the Proposed Method

In previous studies [28,29], they only performed differential expression analyses. The differentially expressed miRNAs were not necessarily good biomarkers. In fact, there were usually too many differentially expressed miRNAs, and they were highly redundant, which rendered them not suitable as biomarkers. The machine learning methods we proposed, especially the feature ranking algorithms, were proven to find the smallest number of biomarkers with the greatest prediction power.

For the limitations, although we have tested our method on two independent datasets (GSE166160 and GSE178246), the sample size was only approximately 400, which is too small and not representative enough for real applications. These identified biomarkers still need to be validated in a larger sample size and patient cohorts infected with various SARS-CoV-2 strains.

## 5. Conclusions

In this study, an advanced machine learning computational framework was designed to differentiate between COVID-19 patients, SARS-CoV-2-negative patients with upper respiratory symptoms, and the normal healthy population. The severity of the disease in individuals, especially in COVID-19 patients, was further determined. Four feature ranking algorithms (LASSO, LightGBM, MCFS, and mRMR) were used to filter out the miRNAs with significant impacts on the classification of the patients. Some miRNAs affecting SARS-CoV-2 infection, such as miR-24-3p, miR-93-3p, and miR-148a-3p, were identified, indicating the reliability of the results reported in this study. Subsequently, based on the screened miRNAs, RF, and KNN showed efficient classification performances in the present study. Meanwhile, we extracted the decision process of the DT to form the classification rules, describing the role of miRNA in the classification process at a quantitative level. Eventually, we explained the rationality of the miRNAs and rules for distinguishing the different types of patients through the literature, thereby reflecting the reliability and efficiency of this research.

## Figures and Tables

**Table 1:** Overall performances of the optimal classifiers under different classification algorithms and feature lists yielded with different feature ranking algorithms.

Feature Ranking Algorithm	Classification Algorithm	Number of Features	ACC	MCC	Macro F1	Weighed F1
LASSO	DT	1335	0.680	0.595	0.710	0.677
	KNN	734	0.747	0.700	0.788	0.733
	RF	300	0.739	0.686	0.779	0.725
	SVM	406	0.709	0.644	0.759	0.700
LightGBM	DT	418	0.699	0.618	0.727	0.698
	KNN	834	0.760	0.711	0.801	0.751
	RF	114	0.787	0.741	0.812	0.780
	SVM	275	0.731	0.673	0.769	0.723
MCFS	DT	639	0.696	0.616	0.733	0.694
	KNN	55	0.747	0.689	0.780	0.741
	RF	239	0.757	0.707	0.794	0.748
	SVM	381	0.720	0.659	0.762	0.709
mRMR	DT	249	0.677	0.594	0.719	0.673
	KNN	823	0.747	0.699	0.787	0.735
	RF	583	0.749	0.699	0.788	0.741
	SVM	141	0.720	0.659	0.762	0.713

**Table 2:** Performances of the models on essential feature sets yielded with different feature ranking algorithms.

Feature Ranking Algorithm	Classification Algorithm	Number of Features	ACC	MCC	Macro F1	Weighed F1
LASSO	KNN	129	0.707	0.645	0.740	0.696
LightGBM	RF	62	0.771	0.721	0.803	0.764
MCFS	RF	75	0.731	0.679	0.774	0.714
mRMR	RF	39	0.725	0.669	0.768	0.712

**Table 3:** Summary of the representative miRNAs associated with COVID-19 severities.

miRNA	Target Gene	Expression Level	Predicted Class	Ref.
miR-24-3p	NRP-1	Upregulated	Healthy	[53,54]
miR-93-3p	TLR4	Upregulated	Severe COVID-19	[55,56]
miR-148a-3p	SOS2, BACH2, MITF	Upregulated	Severe COVID-19	[57,58]
miR-139-5p	MYD88, c-FOS, RAP1B	Downregulated	Non-COVID-19-mild	[59,60]
miR-199a-5p	ARHGAP21	Upregulated	Healthy	[61,62]
miR-17-3p	NIBP	Upregulated	Severe COVID-19	[54,63,64,65,66]
miR-200c-3p	ACE2, IL8	Downregulated	Non-COVID-19-severe	[67,68,69,70,71]
miR-6750-5p	POU2F2	Downregulated	Severe COVID-19	[72]
miR-93-5p	PDCD1LG2	Downregulated	Severe COVID-19	[73]
miR-34a-5p	SDK2	Upregulated	Severe COVID-19	[61,74,75,76,77]
miR-29b-2-5p	POU2F2	Upregulated	Severe COVID-19	[78]
miR-429	NR5A2	Downregulated	Severe COVID-19	[79,80]
miR-205-5p	MOSMO	Downregulated	Severe COVID-19	[81,82,83]
miR-873-5p	PHF6	Downregulated	Severe COVID-19	[84]

## Data Availability

The data presented in this study are openly available in Gene Expression Omnibus at https://www.ncbi.nlm.nih.gov/geo/ (accessed on 15 May 2022), reference number [28,29].

## Supplementary Materials

The following supporting information can be downloaded at: https://www.mdpi.com/article/10.3390/life12121964/s1, Table S1: Feature ranking results obtained using LASSO, LightGBM, MCFS, and mRMR; Table S2: Performance of IFS with different classification algorithms. Columns 2 to 7 represent the F1 scores on different classes. Columns 8 to 11 represent the overall performance measurements; Table S3: The intersection results of the essential feature sets extracted from four feature lists generated by LASSO, LightGBM, MCFS, and mRMR; Table S4: Classification rules generated by the optimal DT model. Each rule is comprised of miRNA features and their expression values. It describes how the feature’s high or low expression value affects the ability to distinguish among various classes of samples.
